# In vitro total phenolics, total flavonoids, antioxidant and antibacterial activities of selected medicinal plants using different solvent systems

**DOI:** 10.1186/s13065-022-00858-2

**Published:** 2022-08-27

**Authors:** Ansar Mehmood, Sonia Javid, Muhammad Faraz Khan, Khawaja Shafique Ahmad, Amna Mustafa

**Affiliations:** Department of Botany, University of Poonch Rawalakot (UPR), Rawalakot, Azad Kashmir 12350 Pakistan

**Keywords:** Total phenolic content, Antioxidant, Antibacterial, Solvent systems

## Abstract

Recently, an interest has surged in utilizing indigenous medicinal plants to treat infectious illnesses and extract bioactive substances, highlighting the need to analyze medicinal plants for phytochemicals and bioactivities. The present study was aimed to evaluate the impact of different solvent systems (aqueous, ethanol, and methanol) used for extraction on total phenolics, total flavonoids, antioxidant, and antibacterial activity of three medicinal plants of Azad Kashmir (*Achillea millefolium*, *Bergenia ciliata,* and *Aloe vera*). High phenolic content was found in methanol extracts of *B. ciliata* (27.48 ± 0.58 mg GAE/g dry weight), *A. vera* (25.61 ± 0.33 mg GAE/g dry weight), and *A. millefolium* (24.25 ± 0.67 mg GAE/g dry weight). High flavonoid content was obtained in the ethanol extract of *A. millefolium* (27.13 ± 0.64 mg QE/g dry weight), methanol extract of *B. ciliata* (17.44 ± 0.44 ± 0.44 mg QE/g dry weight), and the methanol extract of *A. vera* (14.68 ± 0.67 mg QE/g dry weight). Strong 2,2-diphenyl-1-picrylhydrazyl radical scavenging activity (DPPH) was obtained with a methanol extract of *B. ciliata* (IC_50_ = 60.27 ± 0.20 µg/mL). With a zone of inhibition and a minimum inhibitory concentration ranging from 10.00 ± 0.66 to 24.67 ± 1.21 mm and 78 to 625 µg/mL, respectively, all of the studied plants demonstrated notable antibacterial activity against *Staphylococcus aureus* and *Escherichia coli*. *A. vera* showed greater antibacterial activity as compared to other plants under study while methanolic extract showed greater antibacterial activity than ethanolic and aqueous extract. The findings of this research support the use of these medicinal plants to treat a variety of diseases.

## Introduction

The use of herbal plants for the treatment of various human ailments has a long history in many civilizations [1–4]. According to some estimates, 75% of people worldwide rely on indigenous medicine [5]. The global health organization (WHO) also notes that a significant section of the inhabitants in underdeveloped countries continue to rely on medicinal plants as their primary sources of healthcare [6]. Because of a variety of factors, including expensive pricing and the inaccessibility of contemporary synthetic pharmaceuticals, people of rural regions chose herbal plants as their major source of health care. Few or no side effects of herbal medicines are reported as compared to the allopathic medics [7]. The plants have a wide range of bioactive substances such as alkaloids, flavonoids, lignins, tannins, terpenoids, polyphenols, vitamins, and other secondary metabolites, making them potential antioxidant and antibacterial agents [8–10]. The bioactive compounds in plants act significantly on parasitic and pathogenic organisms [11–13]. The polyphenols, flavonoids and other associated secondary metabolites significantly reduce the damaging effect of reactive oxygen species (ROS) which are resulted from oxidative stress and cause many human illnesses such as cardiac, degenerative disorders, diabetes, and cancer [14]. Although various antibiotics have been formulated for the treatment of infectious diseases and it was expected that infectious diseases will be eradicated after the discovery of antibiotics [15]. But the fight against the infectious diseases is progressively getting weaker because of antibiotic resistance [16, 17]. In this context, the evaluation of medicinal plants for phytochemicals, antioxidant, and antibacterial activity is necessary for finding new possible therapeutic compounds [13]. Indeed, one fourth of all synthetic drugs used today were made up of any plant-based compound as the active ingredient [18]. The herbal medicines have advantages of no or minimal side effects as compared to synthetic antibiotics [19]. Identification and isolation of target bioactive compounds had been a challenge during entire era of modern pharmacognosy. One major bottle neck has been the selection of appropriate solvent system for crude extract preparation and selection of vehicles for bioassays [20]. Appropriation of solvent system thus, is highly selective and needs considerable attention for better exploration of chemical diversity of plant extract under examination. One plausible approach is to use a panel of most commonly used solvents as a variable during extract preparation and bioassays [21, 22].

Traditional medicines (TM) are commonly utilized in India, Pakistan, China, Korea, Thailand, Japan and Sri Lanka. More than 75% of Pakistanis use TM for all or most of their medical needs [23]. Approximately 600 plant species are utilized medicinally in Pakistan [24]. In Azad Kashmir, there are many plant species which are being used for the treatment of various human diseases. Traditional medicine is heavily used in this region to satisfy people’s daily health care requirements. For example, *A. millefolium*, *B. ciliata*, and *A. vera* are the most popular plants used by the local inhabitants and traditional healers for the treatment of different diseases. *A. vera*, belongs to family Xanthorrhoeaceae, is an herb found all over the world. It is found that it has noticeable pharmacological activities such as antioxidant [25–27], antibacterial [28, 29], anti-aging and anti-inflammatory [30, 31]. Since long time it is used as an herbal drug containing more than 75 bioactive components [32]. *A. vera* holds health supporting effect and is a major source of several phytochemicals like amino acids, salicylic acids, minerals, polysaccharides, anthraquinones, saponins, vitamins and may more [33, 34].

The *Achillea* genus, belonging to the Asteraceae family, is characterized by about 85 species [35]. In many applications, *A. millefolium* (common yarrow) has been used in cosmetics, medicine, and veterinary [36]. As a result, this flowering herb has been used to treat rheumatism, colds, flatulence, and hysteria, and it is said to have antispasmodic, tonic, diaphoretic, and vulnerary properties [37]. *B. ciliata* (Saxifragaceae) is one of the most important commonly used medicinal plants and is known as “Paashanbheda” (meaning ‘to dissolve the stone’). In herbal preparation, *B. ciliata* rhizome has been used for centuries. In the Indian Ayurvedic system, it is used for the suspension of bladder and kidney stones. In south, central, and east Asia, it is very commonly distributed in temperate regions (Kashmir to Nepal) from 2000 to 2700 m. It has been reported from the Kolahai Mountains and the Pir Panjal region in the Kashmir Himalaya [38].

As far as we know, there has not been any study about the phytochemical, antioxidant, and antibacterial activity of these medicinal plants of Azad Kashmir using different solvent systems. Therefore, in this study, total phenolic contents, total flavonoid contents, antioxidant activity, and antibacterial activity of *A. millefolium*, *B. ciliata*, and *A. vera* extracts prepared with different solvents (aqueous, ethanol, and methanol) were studied.

## Methods/experimental

### Plant collection and identification

The plant parts such as leaves of *A. millefolium*, rhizome of *B. ciliata*, and leaves of *A. vera* were collected from different localities of Azad Kashmir. The plant specimens were identified by comparing them to the herbarium specimens deposited in the Department of Botany’s herbarium at the University of Poonch Rawalakot, as well as by using the flora of Pakistan. The scientific names were confirmed by The Plant List (http://www.theplantlist.org). The voucher specimen of each plant was submitted to in the Herbarium of the Department of Botany, University of Poonch Rawalakot. The plant names, voucher numbers, plant parts, and ethnobotanical uses are given in Table 1.

**Table 1:** The scientific names, voucher numbers, plant parts, and ethnobotanical uses of plants analyzed in this study

Plant name	Voucher number	Plant part	Ethnobotanical uses
*Aloe vera*	842-UPR-Poonch	Gel and latex	Used as wound and burn healing, immunomodulatory, gastro-protective, antifungal, anticancer, hypoglycemic, and anti-inflammatory properties [39]
*A. millefolium*	881-UPR-Poonch	Stem, leaves, and flowers	The plant is known for the treatment of inflammation, pain, and gastrointestinal disorders [40]
*B. ciliata*	722-UPR-Poonch	Rhizome	The rhizome of the plant is used in gastrointestinal disorders and for the treatment of kidney stones [41]

### Preparation of plant extracts

Plant extract was prepared using plant parts such as *A. millefolium* leaves, *B. ciliata* rhizome, and *A. vera* leaves. The plant components were dried thoroughly in the shade before being processed into a fine powder with an electric grinder. The powder (100 g) of each plant sample was effectively extracted using aqueous, ethanolic, and methanolic solvent systems via maceration procedure. The macerate of each plant was filtered using Whatman filter paper to separate liquid and solid fractions. To get crude extract, the filtrate was evaporated using a rotary evaporator at reduced pressure. For further investigation, the crude extracts were kept at – 20 °C. The yield extraction of each extract was calculated by using the equation, yield (%) = (weight of extraction/weight of dry sample) × 100.

### Development of different solvent systems

The choice of solvents for extraction is a marriage between their bulk physicochemical properties and their capability to dissolve compounds because they possess complementary intermolecular interactions to the target compounds [42]. For comparison of bioactivity potential of selected plant extract, a total of two organic (methanol and ethanol) and an aqueous solvent systems were used. Despite of a couple of serious efforts for standardization in selection of solvents [43–45], major bulk of studies reporting bioactivities of medicinal plants use any of these solvents arbitrarily. Herein, we have used the choice of solvent system as a variable for a given plant. This is very likely to answer the questions arising from unexpected gain or loss of predicted bioactivities, potentially due to use of inappropriate solvent systems [46, 47].

### Total phenolic content (TPC)

Using gallic acid as a reference, the total phenolics in each plant extract of the examined plants were measured by the Folin-Ciocalteu spectrophotometric technique [48]. In brief, 20 µL of plant extract (10 mg/mL) was combined with 100 µL of Folin-Ciocatleu reagent and vortexed. This mixture was vortexed again after 80 L of 7.5% Na_2_CO_3_ was added, followed by a 30-min incubation period at 45 °C. The absorbance was then measured at 750 nm using a UV–visible spectrophotometer. Using a gallic acid calibration curve, the TPC was calculated as mg GAE/g dry weight of material.

### Total flavonoid content (TFC)

For the measurement of total flavonoids in each plant extract, 24 μL of plant extract (10 mg/mL) was mixed with 28 µL of NaNO_2_ (50 g/L) and kept for 5 min. After that, 28 µL of AlCl_3_ (100 g/L) was added and the mixture was allowed to react for 6 min. Then, 120 µL of 1 M NaOH was added to the mixture, and the absorbance was immediately measured at 510 nm. The TFC was calculated as mg QE/g dry weight [49].

### Antimicrobial assay

For antibacterial activity, each plant extract was dissolved in 0.2% dimethyl sulfoxide (DMSO) at a concentration of 100 mg/mL (100 mg crude extract in 1 mL of DMSO. The antibacterial activity was assessed through a disc diffusion assay using the protocol of the Clinical and Laboratory Standards Institute (CLSI) [50]. Three extracts (aqueous, ethanol, and methanol) of each plant were tested against a gram positive (*S. aureus*) and a gram negative (*E. coli*) bacterium. The viability of each bacterium was tested by culturing them in nutrient broth under sterile conditions. After that, the viable strains were sub-cultured on nutrient agar medium and incubated at 37 °C for 24 h. Fresh microbial cultures were produced from this stock culture and cultivated for 24 h at 37 °C in nutrient broth medium. The final counts of bacteria were maintained to a 0.5 McFarland turbidity standard (1.5 × 108 CFU/mL) using sterile saline solution. Muller Hinton agar was put in Petri dishes and the bacterial suspension was spread over them with the help of a cotton swab. Sterile discs made of Whatman no. 42 filter paper were immersed for 5 min in 50 µL of crude extract solution before being placed on the surface of Petri plates containing test bacteria and incubated for 24 h at 37 °C. The diameter of the zone of inhibition around the disc was measured in millimeters after 24 h. As negative and positive controls, 50 µL of 0.2% DMSO and a gentamicin antibiotic disc were used in the same manner as plant extract. All the activities were performed in triplicate.

Further, minimum inhibitory concentration (MIC) broth dilution was also used to determine the MIC of each plant extract. The serial dilution of crude extracts was made as 2500, 1250, 625, 312, 156, 78, 39, and 19 μg/mL. In test tubes, different concentrations of crude extracts, 10 mL nutrient broth samples, and 100 μL of active inoculums of bacterial suspension (1.5 × 108 CFU/mL) were inoculated and then incubated at 37 °C for 24 h. The MIC was defined as the lowest concentration of plant extract that inhibited bacterial growth. The absence of turbidity and inoculation on agar containing Petri dishes proved the lack of growth.

### Antioxidant activity by DPPH assay

The antioxidant activity of each extract of tested plants was measured by the DPPH scavenging assay with a few modifications [51]. All the plant extracts were dissolved and homogenized in 95% methanol with a final concentration of 50 mg/mL. The stock solution is diluted into 5 concentrations (10, 20. 30, 40, and 50 mg/mL). The same concentrations were used for l-ascorbic acid, which serves as a positive control. In 95% methanol, a 0.2 mM DPPH solution was also prepared. Briefly, an equal volume of sample and DPPH solution was added to maintain a 4 mL final volume. The control was prepared in the same manner by replacing the sample with methanol. All of the samples were vortexed before being incubated in the dark for 30 min at 37 °C. The reaction samples were prepared in triplicates. Using a UV spectrophotometer, the absorbance of each sample was measured at 517 nm. The following formula was used to calculate the percentage of DPPH scavenging activity.

$$\mathrm{DPPH\, inhibition }({\%})=\frac{\mathrm{Absorbance\, of\, control}-\mathrm{Absorbance\, of \,sample}}{\mathrm{Absorbance\, of \,control}}\times 100$$

The results were reported as an IC_50_ value, which was obtained by plotting DPPH percentage inhibition versus log concentration of sample extract. A lower IC_50_ value indicates higher DPPH scavenging ability.

### Statistical analysis

All the experiments were conducted in triplicates and results are represented as the means and standard errors of the means. For antibacterial data, one way ANOVA was performed, and means were compared through Least Significant Difference by using Statistix 8.1. Significant level was defined as P ≤ 0.05.

## Results and discussion

### Extraction yield

Percentage yield of crude extracts was calculated for all three types of solvents (Table 2). Despite of some deviations, methanol remained the best solvents with an extraction yield range of 6.10 ± 0.44 to 33.65 ± 0.61%. *B. ciliata* was the best extracted plant followed by *A. vera* and *A. millefolium*. Better extraction with methanol might be linked to the fact that methanol has a better penetration efficacy across the plasma membranes that facilitates immediate liberation of cellular contents into the solvent and hence results in net higher yield of crude extract. Our findings on better yield and subsequent bioactivities of methanolic extract is augmented by previous reports on bioassays in differential solvent systems [52, 53].

**Table 2:** Extraction yield, total phenolic content (TPC), total flavonoid content (TFC), and antioxidant activity (in IC_50_) of extracts (aqueous, ethanol, and methanol) of *A. millefolium*, *B. ciliata*, and *A. vera*

Plants	Extracts	Yield (% w/w)	TPC (mg GAE/g sample)	TFC (mg QE/g sample)
*A. millefolium*	Aqueous	6.10 ± 0.44	16.34 ± 0.71	12.96 ± 0.67
	Ethanol	5.18 ± 0.28	20.32 ± 0.55	25.67 ± 0.55
	Methanol	14.25 ± 0.67	24.25 ± 0.67	27.13 ± 0.64
*B. ciliata*	Aqueous	12.10 ± 0.44	18.26 ± 0.71	12.79 ± 0.34
	Ethanol	15.16 ± 0.71	21.44 ± 0.60	16.68 ± 0.58
	Methanol	33.65 ± 0.61	27.48 ± 0.58	17.44 ± 0.44
*A. vera*	Aqueous	12.72 ± 0.45	16.75 ± 0.60	12.91 ± 0.58
	Ethanol	15.68 ± 0.39	17.13 ± 0.67	11.31 ± 0.20
	Methanol	21.67 ± 0.67	25.61 ± 0.33	14.68 ± 0.67

### Total phenolic contents (TPCs) and total flavonoid contents (TFCs)

The extracts were screened for the total contents of phenolics and flavonoids that may have contributed to their antioxidant and antibacterial activities. The results are shown in Table 2. It was noted that methanol extracted more phenolic and flavonoid compounds as compared to other solvent systems. The TPC in methanolic extract was 27.48 ± 0.58, 25.61 ± 0.33 and 24.25 ± 0.67 mg GAE/g dry weight for *B. ciliata*, *A. vera* and *A. millefolium* respectively. Similarly, the TFCs in methanolic extract of *A. millefolium*, *B. ciliata*, and *A. vera* were 27.13 ± 0.64, 17.44 ± 0.44, and 14.68 ± 0.67 mg QE/g sample, respectively. The higher TPCs and TFCs in methanolic extract can be attributed to the shorter size of methyl radical as compared to the ethanol [54]. Many studies have reported higher level of TPCs and TFCs in methanolic extract of the plants, owing to more polar nature of methanol than other organic solvents. Truong et al. [22] found higher phenolic, flavonoid and alkaloid contents in methanolic extracts of *Severinia buxifolia*. Certainly, the efficacy and throughput of extraction is largely affected by various optimization factors such as retention time, temperature and grade of chemicals [55, 56]. Traditionally, *B. ciliata* is known for removal of kidney stones and is called “Pather chatt” in Kashmir Himalayas. It is also reported for antioxidant, antibacterial and antimalarial effect. The plant is also reported to have high phenolic contents [49]. Our findings are in agreement with previous reports and highlight the significance of solvent system selection for better pharmacological reproducibility.

Phenolic substances are secondary metabolites present in plants having a variety of biological functions, including protection from oxidative damage [57–59]. Plants have been shown to produce phenolic chemicals in response to oxidative stress [60, 61]. On the other hand, flavonoids, a class of phenolic compounds are well known for antioxidant property [62]. Besides acting as antioxidants, flavonoids function as stabilizers of scavenging chemicals that are otherwise washed away by ROS flooding. This dual role is acclaimed to large quantity of free OH groups, notably 3-OH and resultant higher reactivity of flavonoids hydroxyl groups with oxidants [63, 64].

### Antioxidant activity

**Fig. 1:**
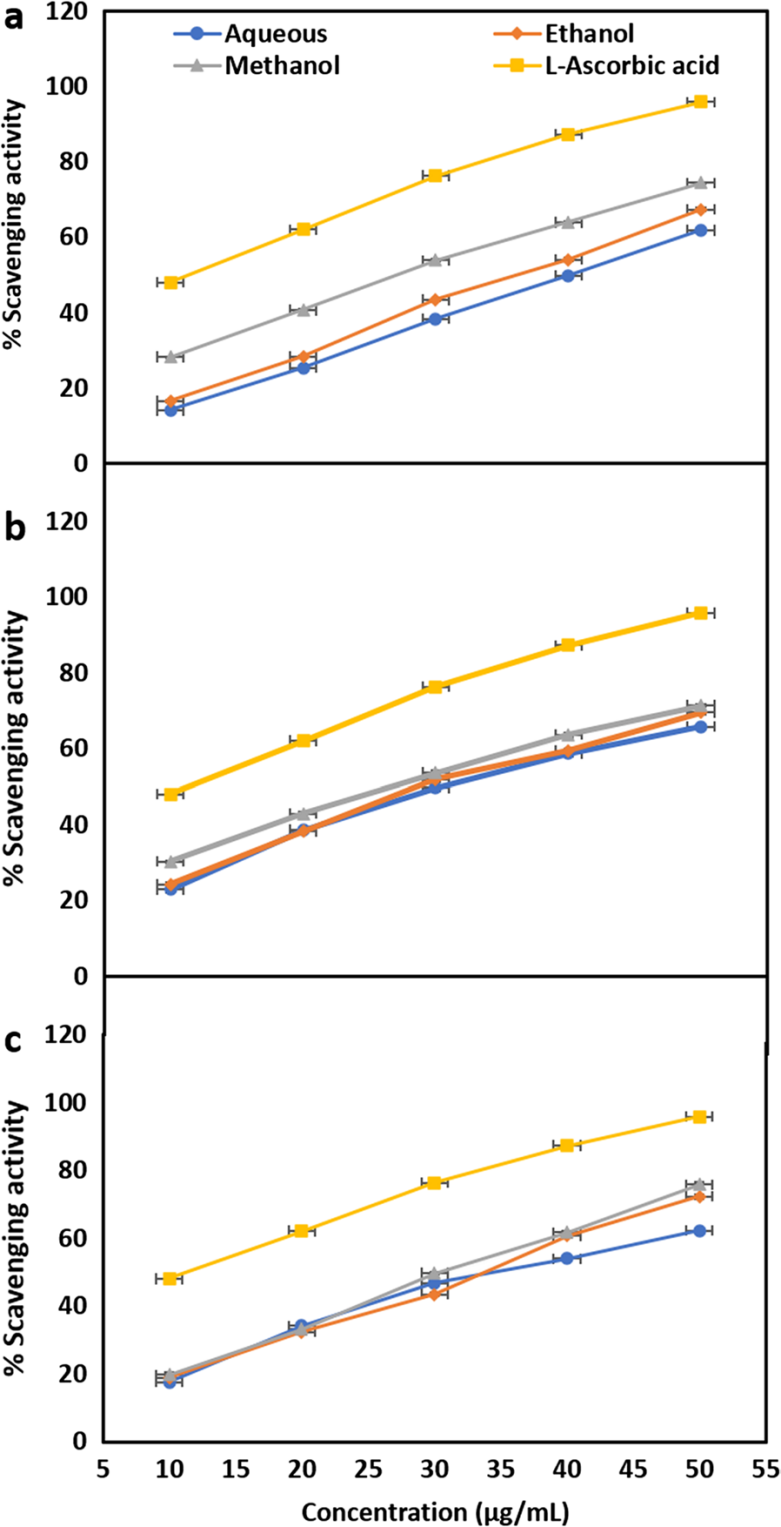
Concentration dependent DPPH scavenging activity of extracts, **a** extracts of *A. millefolium*, **b** extracts of *B. ciliata*, and **c** extracts of *A. vera*

**Fig. 2:**
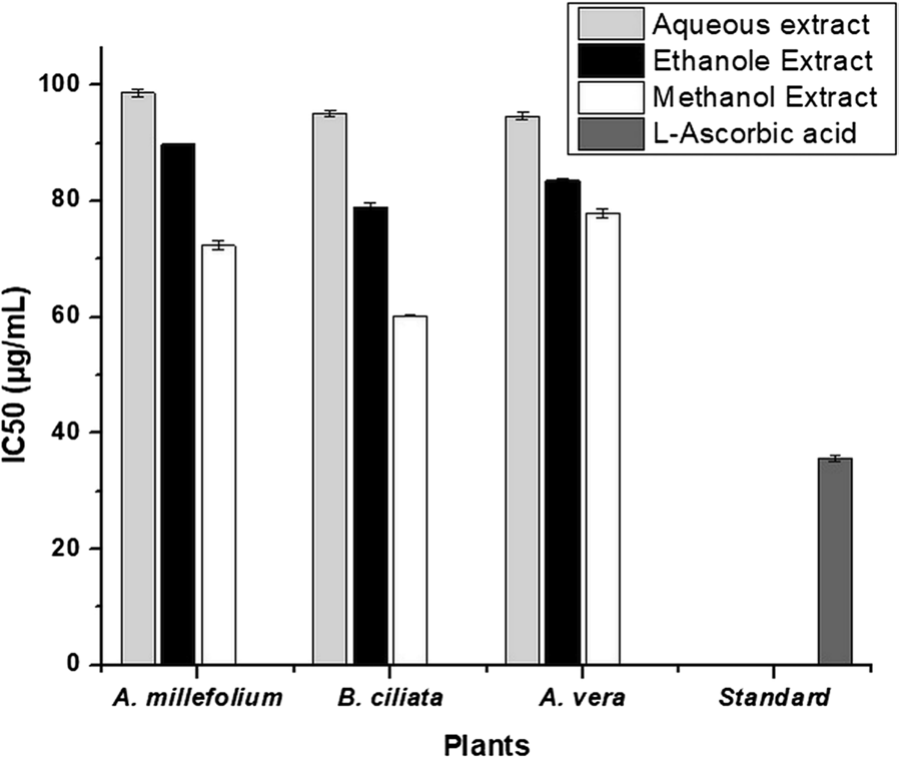
IC_50_ values of plant extracts and ascorbic acid (standard) against DPPH radicals, bar represents the mean value of 3 replicates, error bar represents the standard errors of means

The antioxidant activity of the extracts was determined using DPPH assay. The capabilities of the extracts of *A. millefolium*, *B. ciliata*, and *A. vera* prepared with various solvents to scavenge DPPH free radicals compared to the capability of ascorbic acid at different concentrations ranging from 10 to 50 µg/mL are shown in Fig. 1. The DPPH scavenging activity of various extracts was concentration dependent, increased with an increase in the concentration of extracts. Among the extracts, the methanolic extract of each test plant showed the highest antioxidant activity. The IC_50_ values recorded for methanolic extract were 72.33 ± 0.73, 60.27 ± 0.20, and 77.89 ± 0.67 µg/mL, respectively for *A. millefolium*, *B. ciliata*, and *A. vera* (Fig. 2). The methanol extract of *B. ciliata* had the strongest antioxidant activity among the plants examined, with an IC_50_ value that lowest and closest to standard l-ascorbic acid (35.66 0.58 µg/mL). The highest antioxidant activity of methanolic extract hints its relationship to more polar constituents, as also reported in previous studies [65]. These polar compounds might be bergenin, gallic acid, tannic acid, catechin, ()-3-*O*-galloylcatechin, and [10]- 3-O-galloylepicatechin that are present in the rhizome of *B. ciliata* [66, 67]. In addition to these, many other secondary metabolites such as polyphenols, carotenoids and alkaloids have been reported for significant antioxidant activity [68]. Higher antioxidant activity of the plant extract can also be linked to other electron donors in the extract that might react with free radicals and transform them to more stable products thereby disrupting the free radical chain [69].

Furthermore, we developed a Pearson correlation between the TPC, TFC, and antioxidant activity of test plants to find the relationship between these biocompounds and antioxidant activity of the plants (Table 3). A highly positive correlation was found between the TPC and antioxidant activity of all the test plants. Due to this positive relationship, it can easily be predicted that the higher antioxidant activity of methanolic extract was due to higher amount of TPC in methanolic extract. Our results are in agreement with the previous studies that TCP and antioxidant activities are strongly correlated [70, 71], because TPC is a significant antioxidant agent found in many medicinal plants, including vegetables and fruits [72]. The methanolic extract of sea buckthorn also showed strong antioxidant activity [73]. The oxidoreduction characteristics of phenolic compounds contribute to their antioxidant capacity by acting as hydrogen donors, reducing agents, possible metal chelators, and singlet oxygen quenchers [74, 75]. The hydroxyl groups in phenolics and their derivatives react with ROS and reactive nitrogen species in a termination reaction, breaking the process of fresh radical formation [76–78].

**Table 3:** Pearson correlation between the antioxidant activity and total phenolic content

	*A. millefolium*			*B. ciliata*			*A. vera*		
	TPC	TFC	DPPH	TPC	TFC	DPPH	TPC	TFC	DPPH
TPC	1		0.997*	1		1.000**	1		0.727
TFC		1	0.817		1	0.917		1	0.357
DPPH			1			1			1

TPC: Total phenolic content, DPPH: 2,2-diphenyl-1-picrylhydrazyl

*Significant at *p* < 0.05, **significant at *p* < 0.001

### Antibacterial activity

We have tested three extracts (aqueous, ethanol, and methanol) of *A. millefolium*, *B. ciliata*, and *A. vera* against gram positive and gram-negative bacterium and found them to have promising antibacterial activity against both types of bacteria, with a zone of inhibition ranging from 10.00 ± 0.66 to 24.67 ± 1.21 mm (Table 4). The extracts also showed noteworthy MIC values ranging from 78 to 625 µg/mL. Previous studies reported a wide range of MICs for plant derived natural products. The previous study suggested the range of MIC value between 156 and 625 µg/mL as a good antibacterial activity [79]. It has also been suggested that isolated phytochemicals should have an MIC value of less than 780 µg/mL [80]. Similarly, Kuete [81] marked the significant MIC values by reviewing the literature as an MIC values less than 100 µg/mL are significant, between 100 and 625 µg/mL is good, and more than 625 µg/mL is low. In our study, we considered 78 to 312 µg/mL MIC values as significant antibacterial activity and highlighted them in bold (Table 5).

**Table 4:** Antibacterial activity of extracts (aqueous, ethanol, and methanol) of *A. millefolium*, *B. ciliata*, and *A. vera* (mean values of zone of inhibition (in mm) including the diameter of disc 6 mm) against *S. aureus* and *E. coli*

Plants	Bacteria	The extracts (at the concentration of 100 mg/mL DMSO)				
		Aqueous	Ethanol	Methanol	DMSO	Gentamycin (100 mg/mL DMSO)
*A. millefolium*	*S. aureus*	20.00 ± 1.05^c^	23.00 ± 1.6^b^	24.33 ± 1.21^b^	nt	31.33 ± 1.21^a^
	*E. coli*	10.00 ± 0.66^b^	23.67 ± 1.5^a^	11.00 ± 0.66^b^	nt	23.67 ± 1.60^a^
*B. ciliata*	*S. aureus*	20.33 ± 1.01^c^	20.33 ± 2.33b^c^	24.00 ± 1.03^b^	nt	31.33 ± 1.21^a^
	*E. coli*	17.67 ± 0.66^b^	15.33 ± 0.58^b^	23.67 ± 2.14^a^	nt	23.67 ± 1.60^a^
*A. vera*	*S. aureus*	15.67 ± 1.60^c^	20.67 ± 1.60^b^	27.67 ± 1.21^b^	nt	31.33 ± 1.21^a^
	*E. coli*	14.00 ± 1.05^c^	17.66 ± 0.60^b^	22.33 ± 1.40^a^	nt	23.67 ± 1.60^a^

nt: not detected. Different letters in a row indicate the significant different between the values at *p* = 0.05 by least significant difference test

**Table 5:** Minimum inhibitory concentration of extracts (aqueous, ethanol, and methanol) of *A. millefolium*, *B. ciliata*, and *A. vera* against *S. aureus* and *E. coli*

Plants	Test bacteria	Minimum inhibitory concentration (µg/mL)		
		Aqueous	Ethanol	Methanol
*A. millefolium*	*S. aureus*	**≥ 156**	**≥ 78**	**≥ 78**
	*E. coli*	≥ 625	**≥ 156**	≥ 625
*B. ciliata*	*S. aureus*	**≥ 312**	**≥ 156**	**≥ 78**
	*E. coli*	**≥ 156**	**≥ 312**	**≥ 78**
*A. vera*	*S. aureus*	**≥ 312**	**≥ 312**	**≥ 78**
	*E. coli*	**≥ 156**	**≥ 312**	**≥ 156**

The bold emphasis indicates the significant MIC values

A significant difference in antibacterial activity was found when *A. millefolium* was extracted with different solvents (aqueous, ethanol, and methanol), ranging from 20.00 ± 1.05 to 24.33 ± 1.21 mm against *S. aureus* and 10.00 ± 0.66 to 23.67 ± 1.50 mm against *E. coli.* The highest activity was shown by methanolic extract against *E. coli* (24.33 ± 1.21 mm). The ethanol extract also showed significant activity against both the tested bacteria. Previous studies also confirm the significant antibacterial activity of methanolic extract of *A. millefolium* [82–84]. When the different extracts of *B. ciliata* were tested, a pronounced antibacterial activity was observed against the bacterial strains. Again, methanolic extract exhibited the highest growth inhibitory activity (24.00 ± 1.03 and 23.67 ± 2.14 mm) against *S. aureus* and *E. coli*, respectively. Sinha et al. [85] also found growth inhibition by methanol extract of rhizome against *S. aureus* with a zone of inhibition of 15 mm. However, we observed a pronounced antibacterial activity by methanolic extract against *S. aureus* (24.00 ± 1.03 mm). This difference in activity may be due to the collection of plants from different ecological conditions. Aqueous extract also showed a good antibacterial activity against both test bacteria. In a similar study to ours, Singh et al. [13] also reported higher antibacterial activity of methanol extract of *B. ciliata* than the other solvents used. Similarly, the methanolic extract of *A. vera* showed the best results and inhibited the growth of *S. aureus* by a zone of inhibition of 27.67 ± 1.21 mm. This extract was also highly active against *E. coli,* with a zone of inhibition of 22.33 ± 1.40 mm. The aqueous and ethanol extracts of *A. vera* also showed notable antibacterial activity against the tested organisms. The reason for the highest antibacterial activity of *A. vera* might be the presence of certain bioactive compounds. For example, it contains bioactive anthraquinones, a structural analogue of tetracycline. The anthraquinones work like tetracyclines and inhibit the growth of bacteria [32]. Similarly, *A. vera* also contains polysaccharides and the phenol pyrocatechol, which are known to have toxic effects on microorganisms [86, 87].

*Aloe vera* had the highest antibacterial activity among the plants tested for antibacterial activity, followed by *B. ciliata* and *A. millefolium*. In case of solvents used for the extraction of plant parts, methanol was found to be the most appropriate solvent in terms of the antibacterial activity of the crude extracts. Ethanol and aqueous were ranked 2nd and 3rd, respectively. The different antibacterial activities by different plant extracts were more likely due to the presence of different bioactive compounds in test plants, the extraction capacity of the solvents, the sensitivity of the bacterial strains used, and the combined effect of these conditions. Previous studies also confirm these differences in antibacterial activity by different plant extracts [11, 13, 65, 80]. The phytochemical analysis of tested plants showed the higher amount of TFC extracted by methanol, which could be the reason for the higher antibacterial activity of methanol extract. The flavonoids have been reported to have an inhibitory effect on the biosynthesis of nucleic acid and other metabolic processes in microorganisms [88]. Another reason for the strong antibacterial activity of flavonoids is their effect on the permeability of biological membranes [89]. In addition to flavonoids, phenolic compounds also play an important role in the inhibition of bacterial growth. The C3 side chain of phenols lowers the level of oxidation that results in antimicrobial activity [90]. The partial hydrophobic nature of polyphenols also contributes to microbial lethality by inhibiting protease and by interacting with other proteins and carbohydrates [91].

In our study, we also observed that *S. aureus* (gram-positive) was more susceptible to the plant extract than *E. coli* (gram negative). This is explained by the existence of a distinct outer barrier in gram-negative bacteria that prevents extracellular entry into the cell but is absent in gram-positive bacteria [92]. The overall result suggests that the above studied plants could be a great choice in exploring therapeutic lead compounds in the tested plant species. Recently a large number of compounds such as quinones (embelin), anthraquinone (aloe-emodin and chrysophanol), sterols (fucosterol, B-sitosterol, and stigmasterol), and saponins (spirostane and furostane aglycones) [93, 94]. Medicinal plants are being reported for antibacterial activities. Moreover, our findings about the relevance of appropriate solvent systems for biological evaluation could pave new avenues for studies aiming to evaluate medicinal plants.

## Conclusions

In this study, three indigenously used medicinal plants (*A. millefolium*, *B. ciliata*, and *A. vera*) were evaluated for their TPC, TFC, antioxidant, and antibacterial activity. The IC_50_ values showed that the extracts of these plants have good antioxidant activity. Based on the zones of inhibition and MIC values obtained in this study, it is concluded that these plants have antibacterial activity against *S. aureus* and *E. coli*. These efficient antioxidant and antibacterial activities were supported by the presence of a good amount of total phenolic and flavonoid content in the extracts. Methanol extraction of all these species has higher TPC, antioxidant, and antibacterial activity. This study supports the role of these medicinal plants in preventing different ailments and can be helpful for the discovery of new drugs from these plants in the future.

## Data Availability

All the data obtained, and materials investigated in this research are accessible with the corresponding author.
